# Evaluation of the frequency of mutation genes in multidrug-resistant tuberculosis (MDR-TB) strains in Beijing, China

**DOI:** 10.1017/S0950268820003131

**Published:** 2021-01-05

**Authors:** Y. Liu, Y. Sun, X. Zhang, Z. Zhang, Q. Xing, W. Ren, C. Yao, J. Yu, B. Ding, S. Wang, C. Li

**Affiliations:** 1 Department of Bacteriology and Immunology, Beijing Key Laboratory on Drug-Resistant Tuberculosis Research, Beijing Tuberculosis and Thoracic Tumor Research Institute/Beijing Chest Hospital, Capital Medical University, Tongzhou District, Beijing 101149, China; 2 Beijing Changping Center for Tuberculosis Control and Prevention, Changping District, Beijing 102200, China; 3 Central Laboratory, Beijing Research Institute for Tuberculosis Control, Xicheng District, Beijing 100035, China

**Keywords:** Beijing genotype, isoniazid, multidrug-resistant tuberculosis (MDR-TB), *Mycobacterium tuberculosis*, rifampin

## Abstract

The aim of this study was to explore the frequency and distribution of gene mutations that are related to isoniazid (INH) and rifampin (RIF)-resistance in the strains of the multidrug-resistant tuberculosis (MDR-TB) *Mycobacterium tuberculosis* (*M.tb*) in Beijing, China. In this retrospective study, the genotypes of 173 MDR-TB strains were analysed by spoligotyping. The *katG*, *inhA* genes and the promoter region of *inhA*, in which genetic mutations confer INH resistance; and the *rpoB* gene, in which genetic mutations confer RIF resistance, were sequenced. The percentage of resistance-associated nucleotide alterations among the strains of different genotypes was also analysed. In total, 90.8% (157/173) of the MDR strains belonged to the Beijing genotype. Population characteristics were not significantly different among the strains of different genotypes. In total, 50.3% (87/173) strains had mutations at codon S315T of *katG*; 16.8% (29/173) of strains had mutations in the *inhA* promoter region; of them, 5.5% (15/173) had point mutations at −15 base (C→T) of the *inhA* promoter region. In total, 86.7% (150/173) strains had mutations at *rpoB* gene; of them, 40% (69/173) strains had mutations at codon S531L of *rpoB.* The frequency of mutations was not significantly higher in Beijing genotypic MDR strains than in non-Beijing genotypes. Beijing genotypic MDR-TB strains were spreading in Beijing and present a major challenge to TB control in this region. A high prevalence of *katG* Ser315Thr, *inhA* promoter region (−15C→T) and *rpoB* (S531L) mutations was observed. Molecular diagnostics based on gene mutations was a useful method for rapid detection of MDR-TB in Beijing, China.

## Introduction

Tuberculosis (TB), caused by *Mycobacterium tuberculosis* (*M.tb*) complex infection, is a major threat to public health around the world. The World Health Organization (WHO) has estimated that there were approximately 10 million new cases and 1.4 million deaths due to TB in 2019 [1]. China is one of the 30 high multidrug-resistant (MDR)-TB burden countries. The percentage of MDR-TB cases in China was 4.5%, which is beyond the average value (3.3%) of global [1]. Prevention and control of MDR-TB in Beijing, the capital of the People's Republic of China, is a public health issue.

MDR-TB is defined as TB that is caused by *M.tb* resistant to at least the two most powerful anti-TB drugs, isoniazid (INH) and rifampin (RIF) [2]. Resistance to anti-TB drugs in *M.tb* mainly arises from genomic mutations in genes encoding either the drug target or enzymes involved in drug activation [3]. It has been well established that most INH-resistant strains possess mutations in *katG* and/or the promoter region of *inhA* [4–6]. *M.tb* strains resistant to INH usually carry mutations in *katG* gene and in the promoter of *inhA* [7, 8]. INH resistance is most commonly caused by the substitution of a single nucleotide at codon 315 in *katG* [9, 10]. Resistance to RIF in *M.tb* strains arises primarily from mutations in the *rpoB* gene [3, 11, 12]. Mutations located in an 81-bp region of the *rpoB* gene encoding the *β* subunit of RNA polymerase – termed the RIF resistance determinant region (RRDR) occur in more than 95% of RIF-resistant strains [13]. The most common mutations in the RRDR region occur at codons 513, 516, 526 and 531.

The population structure of MDR-TB strains and the distribution of resistance-associated nucleotide alterations among the strains of different genotypes have not yet been reported for Beijing. Likewise, molecular characterisation of INH-resistant and RIF-resistant *M.tb* strains in different genotypes in Beijing remains unknown. The association between the prevalence of Beijing genotype strains and their MDR-TB status in Beijing is also unclear. While numerous studies have indicated that Beijing genotype *M.tb* strains are more likely to get into MDR-TB strains [5, 12], other epidemiological studies suggested that Beijing genotype strains are less likely to develop drug resistance than non-Beijing genotype strains [14, 15]. It has not yet been possible to draw a solid conclusion on this issue. Therefore, it is very important to perform molecular characterisation of Beijing genotype and non-Beijing genotype strains from Beijing, and to clarify the association between the prevalence of Beijing genotype strains and the occurrence of MDR-TB in Beijing.

Here, we have performed a retrospective cohort study to elucidate the molecular characteristics of mutations in genes associated with INH-resistant and RIF-resistant strains in Beijing, and determined the percentage of resistance-associated nucleotide alterations in the strains of different genotypes. Our results provide a broader profile of MDR-TB and are informative for strategies development for MDR-TB control in Beijing.

## Methods

### Study setting and description of population

**Fig. 1.**
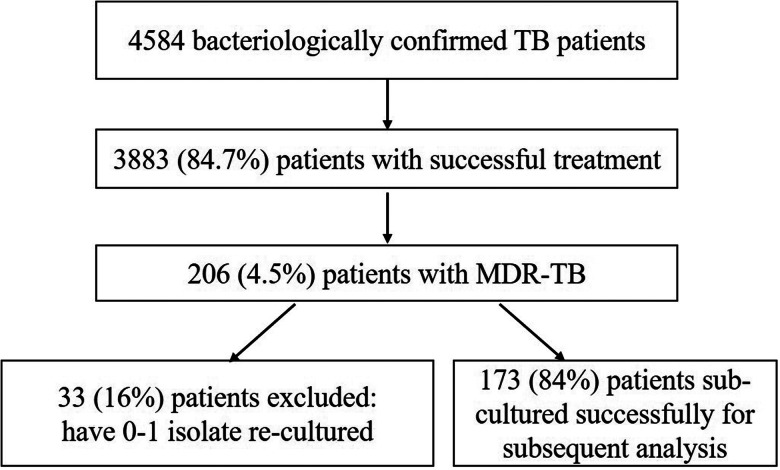
Study population and flowchart outlining the work conducted in this study.

We conducted a retrospective cohort study among local residents and migrant population in Beijing based on strains from a total of 4584 clinical cases that were bacteriologically confirmed (by smear or culture) and reported to the Beijing Research Institute for Tuberculosis Control from 1 January 2008 to 31 December 2010. The median age of the study population was 35 (range 15–81). These patients were epidemiologically unlinked, and all were living in Beijing city. In total, 206 of the 4584 strains were from MDR-TB cases. After recovery on L-J media for 4 weeks at 37 °C, 173 of 206 MDR-TB strains were sub-cultured successfully and submitted for subsequent genotyping and DNA sequencing (Fig. 1). This study was approved by the Ethics Committee of Beijing Tuberculosis and Thoracic Tumor Research Institute. Patients were included when they signed the informed consent form. All the experiments were done in the Department of Bacteriology and Immunology of Beijing Tuberculosis and Thoracic Tumor Research Institute.

### Drug susceptibility testing

Drug susceptibility testing (DST) was performed according to the protocol recommended by WHO/IUATLD [16]. The concentration of anti-TB drugs in Lowenstein-Jensen (L-J) media was as follows: INH 0.2 μg/ml, RIF 40 μg/ml [17]. Strains were considered to show resistance to the specific drug when their growth rate was >1% compared to the control. MDR-TB strains were defined as those strains that were resistant to at least INH and RIF, but not to fluoquinolones (FQs), or any of the three second-line injectable drugs. All drugs were purchased from Sigma-Aldrich (St. Louis, MO, USA).

### Isolation of DNA

Bacterial cells were harvested from L-J cultures, added 400 μl TE buffer (1 M Tris-HCl (pH 8.0) and 0.5 M EDTA (pH 8.0)), incubated for 45 min at 85 °C. The bacterial suspension was incubated with lysozyme (50 mg/ml) at 37 °C overnight, then with 10% SDS and proteinase K (20 mg/ml) at 65 °C for 15 min. DNA was extracted using Cetyltrimethyl Ammonium Bromide (CTAB), followed by phenol extraction and ethanol precipitation [18]. All DNA samples were quantified using a Nanodrop2000 (Thermo Scientific, Wilmington, DE, USA).

### DNA sequencing

The genes coding region of the studied strains was amplified. Primers for each gene were synthesised by Tsingke Co (Beijing, China) and details were listed in Tables S2 and S3. PCR mixtures consisted of template DNA (1 μl), 0.40 μM each of forward and reverse primers, 25 μl 2 × pfu Master mix + loading dye (Generay, Shanghai, China) in a 50 μl reaction volume. PCR products were purified using Axygen PCR purification kits (Axygen, Hangzhou, China) and sequenced (Generay). Mutations were determined by comparison with the H37Rv sequence of each gene in the TBdb database (http://www.tbdb.org/) using BLASTn optimised for megablast on the National Center for Biotechnology Information website (www.ncbi.nlm.nih.gov/BLAST).

### Genotyping

Spoligotyping was carried out using a standard protocol to identify the genotype of *M.tb* strains based on the direct repeat (DR) locus as described previously [19]. Both typical Beijing and Beijing-like genotypes were considered to belong to the Beijing genotype. A commercially available kit (Isogen Bioscience BV, Maarssen, the Netherlands) was used according to the manufacturer's instructions. Membranes were detected with a chemiluminescence system, using ECL detection liquid (Amersham, Buckinghamshire, UK) and X-OMAT film (Kodak, Rochester, NY, USA).

### Statistical analysis

Statistical analyses of genotypes and phenotypes were performed in SPSS 21.0 software. The χ^2^ test or Fisher's exact probability test was used to compare the proportions of different groups. A *P* value <0.05 was considered statistically significant. Odds ratios (ORs) and 95% confidence intervals (CI) were calculated to measure the association relationship between different genotypes and gene mutations.

## Results

### General characteristics of the study population

Strains isolated from 4584 bacteriological positive clinical patients, which has been confirmed active pulmonary TB in Beijing from 1 January 2008 to 31 October 2010. Among these, 3881 (84.7%) strains were from patients who had been cured, and of these, 176 (3.8%) strains were from MDR-TB patients. Three strains were excluded because of difficult re-culture. In total, 173 strains were sub-cultured successfully and submitted for subsequent analysis.

The analysis of the demographic characteristics of the study population (Table S1) indicated that the majority of patients with MDR-TB were male (130 males, 75.1%; 43 females, 24.9%), and the median age was 35 (range from 16 to 80). In total, 101 patients were urban residents of Beijing and 72 were migrant population (non-residents). Ninety-three patients were new TB cases and 80 were retreatment cases. These data suggested that long-term residents of Beijing and the migrant population contributed approximately equally to the prevalence of MDR-TB in Beijing, and that new TB cases and retreated cases were also roughly equivalent. Genotyping of the strains indicated that the Beijing genotype accounted for 90.8% (157/173) of the MDR-TB strains.

### Mutations identified in the *katG* and *inhA* gene

To clarify the molecular characterisation of gene mutations associated with resistance to INH, we sequenced a 2223-bp region of the *katG* gene, encompassing codon 315, and a 520-bp region of *inhA* promoter region of the 173 MDR-TB strains. As anticipated, 79.2% (137/173) of the MDR strains showed mutations in the *katG gene*, 9.8% (17/173) showed mutations only in the *inhA* promoter region, and 17.4% (30/173) showed mutations in both *katG* and the *inhA* promoter region (Tables 1 and 2). Nineteen strains (11%) did not possess mutations in either of the target regions.

**Table 1.** Nucleotide and amino acid changes identified in the specific region of *katG* among 173 MDR-TB strains

Gene	Codon(s)	Nucleotide change	Amino acid change	No. (%) of isolates	Other mutation
*katG* (144)	None	None	None	19 (11)	
	V47V	GTA→GTC	Val	1 (0.6)	
	D90N	GAC→AAC	Asp→Asn	1 (0.6)	
	A93D	GCC→GAC	Ala→Asp	1 (0.6)	
	A110V	GCC→GTC	Ala→Val	1 (0.6)	GCC278ACC (Ala→Thr) in katG
	L141S	TTG→TCG	Leu→Ser	1 (0.6)	
	G156D	GGC→GAC	Gly→Asp	1 (0.6)	
	G184D	GGC→GAC	Gly→Asp	1 (0.6)	
	F185Y	TTC→TAC	Phe→Tyr	1 (0.6)	
	G279R	GGC→CGC	Gly→Arg	1 (0.6)	
	W300S	TGG→TCG	Trp→Ser	1 (0.6)	
	S315N	AGC→AAC	Ser→Asn	3 (1.7)	
	S315N	AGC→AAC	Ser→Asn	1 (0.6)	GAT573TAT (Asp→Tyr) in katG
	S315N	AGC→AAC	Ser→Asn	1 (0.6)	AGC140AAC (Ser→Asn) in katG
	S315N	AGC→AAC	Ser→Asn	1 (0.6)	ATC317GTC (Ile→Val) in katG
	S315T	AGC→ACC	Ser→Thr	87 (50.3)	
	S315T	AGC→ACC	Ser→Thr	1 (0.6)	−8T→C upstream of inhA
	S315T	AGC→ACC	Ser→Thr	1 (0.6)	GGC338GAC (Gly→Asp) in katG
	S315T	AGC→ACC	Ser→Thr	8 (4.6)	−15C→T upstream of inhA
	S315T	AGC→ACC	Ser→Thr	2 (1.2)	GAA582CAA (Glu→Gln) in katG
	S315G	AGC→GGC	Ser→Gly	1 (0.6)	
	Y337S	TAC→TCC	Tyr→Ser	1 (0.6)	
	W412R	TGG→CGG	Trp→Arg	1 (0.6)	
	D573Y	GAT→TAT	Asp→Tyr	1 (0.6)	
	A706E	GCG→GAG	Ala→Glu	1 (0.6)	
	363–368 *del 6bp*			1 (0.6)	
	366–377 *del 12bp*			1 (0.6)	
	371 *del G* and 379 *ins G*			1 (0.6)	
	371 *del G* and 379 *ins G*			1 (0.6)	
	W204stop codon	TTG TAG	Trp→stop code	1 (0.6)

**Table 2.** Nucleotide and amino acid changes identified in the specific region of *inhA* among 173 MDR-TB strains

Gene	Codon(s)	Nucleotide change	Amino acid change	No. (%) of isolates	Other mutation
*inhA*(57)	None	None	None	19 (11)	
		−15C→T		15 (8.7)	
		−15C→T		8	AGC315ACC (Ser→Thr) in katG
		−15C→T		2 (1.2)	CGC145CTC (Arg→Leu) in katG
		−15C→T		2 (1.2)	TAC155TGC (Tyr→Cys) in katG
		−17G>T		1 (0.6)	
		32C＞T		1 (0.6)	
		−15C→T		1 (0.6)	TTC368CTC (Phe→Leu) in katG
		−15C→T		1 (0.6)	ACT380CCT (Thr→Pro) in katG
		−15C→T		1 (0.6)	TAC98TGC (Tyr→Cys) in katG
		−15C→T		1 (0.6)	TAC155TGC (Tyr→Cys) in katG
		−15C→T		2 (1.2)	GGC285AGC (Gly→Ser) in katG
		−15C→T		1 (0.6)	T308P ACC308CCC (Thr→Pro) in katG
		−15C→T		1 (0.6)	CCC92TCC (Pro→Ser), GCC15GGC (Ala→Gly) and AGC315ACC (Ser→Thr) in katG
		−8T→C		1 (0.6)	AGC315ACC (Ser→Thr) in katG

The most frequent mutation (57.2%, 99/173) was a serine to threonine substitution (S315T, AGC→ACC) at codon *katG* 315. Serine to asparagine (S315N, AGC→AAC) substitutions and serine to glycine (S315G, AGC→CGC) substitutions at codon *katG* 315 were also observed in five and one isolates, respectively. Eight strains harboured multisite mutations in *katG*, including one isolate with mutations at codons A110V and A278T, one with mutations at codons D573Y and S315N, one with mutations at codons S140N and S315N, one with mutations at codons I317V and S315N, one with a codon −8 T→C mutation upstream of *inhA* and S315T, one with a codon −15C→T mutation upstream of inhA and S315 T, one isolate with mutations at codons E582Q and S315T, and one with mutation at codons G338N and S315T. In addition, deletions and insertions were also found in *katG* of five MDR strains: at codons 363–368 (6 bp del), 366–377 (12 bp del), 371 (1 bp del, G) and 379 (1 bp ins, G), 371(1 bp del, G) and 379(1 bp ins, G) and a W204stop codon were found. A synonymous mutation (Val47Val) was found within the 593-bp region of the *katG* gene in one isolate.

Thirty-five (20.2%) strains had a C to T substitution at position 15 upstream of the *inhA*. Among them, one had a mutation at codon −8T→C, eight also had a *katG* S315T substitution, and 12 also had other unusual position substitutions. One isolate had −8T→C and a *katG* S315 T substitution (Tables 1 and 2).

When all the *katG* gene and *inhA* promoter mutations were considered, irrespective of if they were single, double or triple, eight genotype patterns emerged. In INH-resistant *M.tb* strains, the most frequently changed codons were S315T (50.3%) and −15C→T (8.7%). The two most frequent mutations accounted for 59% of the INH-resistant strains in this study (Table 3).

**Table 3.** Most frequently identified mutations among the 173 MDR-TB strains

Drug(s)	Locus	Mutation	Other mutation	Frequency (no. of isolates)	Relative frequency (%)
INH	*katG*	Ser315Thr		87	50.3
		Ser315Asn		3	1.7
		Ser315Thr	Glu582Gln in katG	2	1.2
	*inhA*	−15C→T		15	8.7
		−15C→T	Ser315Thr in katG	8	4.6
		−15C→T	Arg145Leu in katG	2	1.2
		−15C→T	Tyr155Cys in katG	2	1.2
			Gly285Ser in katG	2	1.2
RIF	*rpoB*	Ser450Leu		64	37
		Asp435Val		12	6.9
		His445Tyr		12	6.9
		His445Leu		7	4
		His445Asp		6	3.5
		Leu452Pro		6	3.5
		His445Arg		5	2.9
		Leu430Pro		4	2.3
		Asp435Tyr		2	1.2
		His445Tyr	Leu459Arg in rpoB	2	1.2
		Ser450Trp		2	1.2

### Mutations identified in the *rpoB* gene

The *rpoB* gene was sequenced to further characterise MDR-TB strains. Among the 173 MDR strains, 150 (86.7%) isolated strains harboured at least one nucleotide mutation, while 23 (13.3%) strains lacked this mutation within the complete fragment of *rpoB* gene (Table 4).

**Table 4.** Nucleotide and amino acid changes identified in the specific region of *rpoB* gene among 173 MDR-TB strains

Gene	Codon(s)	Nucleotide change	Amino acid change	No. (%) of isolates (173)	Other mutation
*rpoB*	None	None	None	23 (13.3)	
	E312G	GAG→GGG	Glu→Gly	1 (0.6)	CAC445TAC (His→Tyr) in rpoB
	E396V	GAG→GTG	Glu→Val	1 (0.6)	TCG445TTG (Ser→Leu) in rpoB
	L430P	CTG→CCG	Leu→Pro	4 (2.3)	
	L430P	CTG→CCG	Leu→Pro	1 (0.6)	CAC445TAC (His→Tyr) in rpoB
	L430P	CTG→CCG	Leu→Pro	1 (0.6)	GAC435GGC (Asp→Gly) in rpoB
	L430P	CTG→CCG	Leu→Pro	1 (0.6)	GAC435GCC (Asp→Ala) in rpoB
	L430P	CTG→CCG	Leu→Pro	1 (0.6)	ATG434AGG (Met→Arg) in rpoB
	L430P	CTG→CCG	Leu→Pro	1 (0.6)	CAC445TAC (His→Tyr) in rpoB
	L430R	CTG→CGG	Leu→Arg	1 (0.6)	AGC431GGC (Ser→Gly) in rpoB
	L430R	CTG→CGG	Leu→Arg	1 (0.6)	GAC435TTC (Asp→Phe) in rpoB
	Q432K	CAA→AAA	Gln→Lys	1 (0.6)	
	Q432K	CAA→AAA	Gln→Lys	1 (0.6)	AAG799CAG (Lys→Gln) in rpoB
	M434L	ATG→CTG	Met→Leu	2 (1.2)	CAC445TGC (His→Cys) in rpoB
	D435G	GAC→GGC	Asp→Gly	1 (0.6)	
	D435Y	GAC→TAC	Asp→Tyr	2 (1.2)	
	D435G	GAC→GGC	Asp→Gly	1 (0.6)	CAC445GAC (His→Asp) in rpoB
	D435V	GAC→GTC	Asp→Val	12 (6.9)	
	N437D	AAC→GAC	Asn→Asp	1 (0.6)	CTG452CCG (Leu→Pro) in rpoB
	S441L	TCG→TTG	Ser→Leu	1 (0.6)	
	H445D	CAC→GTC	His→Asp	6 (3.5)	
	H445N	CAC→AAC	His→Asn	1 (0.6)	
	H445L	CAC→CTC	His→Leu	7 (4)	
	H445L	CAC→CTC	His→Leu	1 (0.6)	GAC545GAA (Asp→Glu) in rpoB
	H445R	CAC→CGC	His→Arg	5 (2.9)	
	H445Y	CAC→TAC	His→Tyr	12 (6.9)	
	H445Y	CAC→TAC	His→Tyr	1 (0.6)	GAG460GGG (Glu→Gly) in rpoB
	H445Y	CAC→TAC	His→Tyr	2 (1.2)	CTG459CGG (Leu→Arg) in rpoB
	S450L	TCG→TTG	Ser→Leu	64 (37)	
	S450L	TCG→TTG	Ser→Leu	1 (0.6)	CTG459CGG (Leu→Arg) in rpoB
	S450L	TCG→TTG	Ser→Leu	1 (0.6)	CAC835CCC (His→Pro) in rpoB
	S450L	TCG→TTG	Ser→Leu	1 (0.6)	GAC545GAA (Asp→Glu) in rpoB
	S450L	TCG→TTG	Ser→Leu	1 (0.6)	CAC835GAC (His→Asp) in rpoB
	S450L	TCG→TTG	Ser→Leu	1 (0.6)	CTG453ATG (Leu→Met) and CCT483TCT (Pro→Ser) in rpoB
	S450W	TCG→TGG	Ser→Trp	2 (1.2)	
	S450P	TCG→CCG	Ser→Pro	1 (0.6)	
	L452P	CTG→CCG	Leu→Pro	6 (3.5)	
	I491F	ATC→TTC	Ile→Phe	1 (0.6)	
	S493L	TCG→TTG	Ser→Leu	1 (0.6)	ATC491TTC (Ile→Phe) in rpoB

The most common mutation was at codon 531 (*n* = 72, 41.6%), and had three types of amino acid substitutions: S531L (*n* = 69, 39.9%), S531W (*n* = 2, 1.2%) and S531P (*n* = 1). The second most common mutation was at codon 526 (*n* = 35, 20.2%), and five different amino acid substitutions were observed: H526Y (*n* = 15, 8.7%), H526L (*n* = 8, 4.6%), H526D (*n* = 6, 3.5%), H526R (*n* = 5, 2.9%) and H526R (*n* = 1, 0.6%). The third most common mutation was at codon 516 (*n* = 16, 9.25%). The three most frequently observed mutations accounted for 70.5% of the total MDR strains. Furthermore, 11 (*n* = 11, 6.36%) and six (*n* = 6, 3.5%) strains had mutations at codons L511P and L533P, two at codon 513 and two at codon 515, as well as one at codon 288, 477, 518, 522, 572 and 574, respectively. We also detected 14 previously unprotected mutations at codons L511R, Q513K, M515L, D516G, D516V, D516Y, N518D, H526D, H526N, H526L, H526R, H526Y, I572F and S574L, and mutations, E288G and E477V, outside the conventional 81 bp hotspot.

When all the *rpoB* gene mutations were considered, a total of 11 *rpoB* genotype patterns were identified. The most highly mutated codons in RIF-resistance *M.tb* strains were codons 531 (38.2%), 526 (18.5%), 516 (8.1%), 533 (3.5%) and 511 (2.3%). The five most frequently observed mutations accounted for 70.6% of the RIF-resistant strains in this study (Table 3).

### Association between resistance-related mutations and Beijing genotype strains

To clarify the prevalent genotypic diversity of MDR-TB strains in Beijing, we analysed them using spoligotyping method. In total, 157 (90.8%) of the 173 strains belonged to Beijing genotype families and 9.2% (16/173) belonged to non-Beijing genotype families. Statistical analysis of potential associations between gene mutations and the prevalence of strains indicated that Beijing genotype strains did not harbour a higher frequency of gene mutations than non-Beijing genotypes, irrespective of whether the S315T (*P* = 0.529, OR 1.042, 95% CI 0.663–1.637), Ser315N (*P* = 0.554, OR 0.962, 95% CI 0.932–0.992) or *inhA* promoter region including the codon −8T→C (*P* = 0.908) or −15T→C (*P* = 0.589) position were considered. In addition, we did not find significant differences between Beijing and non-Beijing genotypes in the presence of double mutations or no mutations (Table 5).

**Table 5.** Distribution of different mutations conferring INH resistance among Beijing and non-Beijing genotype

Mutation type	Total (*n* = 173)	No. of isolates with different mutations (%)			
		Beijing (*n* = 157)	Non-Beijing (*n* = 16)	OR (95% CI)	*P* value
*katG*					
Ser315Thr	101 (58.4)	92 (58.6)	9 (16.3)	1.042 (0.663–1.637)	0.529
Ser315Asn	6 (3.5)	6 (3.8)	0 (0)	0.962 (0.932–0.992)	0.554
*inhA*					
−8T/C	1 (0.6)	1 (0.6)	0 (0)	0.994 (0.981–0.006)	0.908
−15T/C	35 (20.2)	32 (20.4)	3 (18.8)	1.087 (0.374–3.435)	0.589
Single mutation					
katG	128 (74)	118 (75.2)	10 (62.5)	1.203 (0.814–1.776)	0.208
inhA	13 (7.5)	10 (6.4)	3 (18.8)	0.340 (0.104–1.109)	0.105
Double mutations					
katG + inhA	30 (17.3)	29 (18.5)	1 (6.3)	2.955 (0.431–2.279)	0.193
No mutation					
	12 (6.9)	10 (6.4)	2 (12.5)	0.510 (0.122–2.216)	0.306

The analysis of the distribution of *rpoB* mutation types among different genotype strains indicated that there were no significant differences between Beijing and non-Beijing genotype strains, irrespective of whether mutations at the *rpoB*531, 511, 513, 515, 516, 526 or 533 positions were considered (Table 6).

**Table 6.** Distribution of different mutations located in RDRR of *rpoB* among Beijing and non-Beijing genotypes

Mutation type	Total (*n* = 173)	No. of isolates with different mutations (%)			
		Beijing (*n* = 157)	Non-Beijing (*n* = 16)	OR (95% CI)	*P* value
*rpoB430*					
Leu430Pro	10 (5.8)	8 (5.1)	2 (12.5)	0.408 (0.095–1.758)	0.233
*rpoB432*					
Gln432Lys	2 (1.2)	2 (1.3)	0 (0)	0.987 (0.970–1.005)	0.823
*rpoB434*					
Met434Ile	2 (1.2)	2 (1.3)	0 (0)	0.987 (0.970–1.005)	0.823
rpoB435					
Asp435Val	12 (6.9)	11 (7)	1 (6.3)	1.121 (0.155–8.131)	0.694
Asp435Gly	2 (1.2)	2 (1.3)	0 (0)		
Asp435Tyr	3 (1.7)	2 (1.3)	1 (6.3)	0.204 (0.020–2.126)	0.254
rpoB445					
His445Arg	5 (2.9)	4 (2.5)	1 (6.3)	0.408 (0.048–3.431)	0.388
His445Asp	7 (4)	7 (4.5)	0 (0)	0.955 (0.924–0.988)	0.501
His445Leu	8 (4.6)	7 (4.5)	1 (6.3)	0.713 (0.094–5.438)	0.548
His445Tyr	14 (8.1)	13 (8.3)	1 (6.3)	1.325 (0.185–9.480)	0.621
rpoB450					
Ser450Leu	67 (3.9)	64 (40.8)	3 (18.8)	2.174 (0.771–6.134)	0.069
Ser450Trp	2 (1.2)	1 (0.6)	1 (6.3)	0.102 (0.007–1.553)	0.177
rpoB452					
Leu452Pro	2 (1.2)	1 (0.6)	1 (6.3)	0.102 (0.007–1.553)	0.177
No mutation	19 (11)	14 (8.9)	5 (31.3)	0.285 (0.118–0.689)	0.019

## Discussion

In this study, we performed a detailed analysis and evaluation of the frequency of mutation genes of MDR-TB strains isolated from clinical patients in Beijing, China. We investigated differences in gene mutation between the Beijing and non-Beijing genotypic strains, and analysed the population structure of these MDR-TB patients.

Our data are in agreement with previous studies, which that mutations conferring INH resistance are most frequently detected in the *katG* gene, especially at codon 315 [8, 13]. Most of the 173 MDR-TB strains examined here (89%, 154/173) contained mutations at *katG* and/or the *inhA* promoter region. Substitution of a single nucleotide at codon 315 of the *katG* gene was the most frequently identified mutation type, conferring resistance in approximately 70% of the INH-resistant strains. The result had a similar frequency to that reported for Jiangxi, China (65.8%), and Shanghai, China (72.7%) [20], although it was lower than that reported for Fujian, China [8] (82.7%), Brazil [21] (87.1%) and Russia (93.6%) [22, 23].

It has previously been reported that 10–28% of INH-resistant strains have a −15C→T mutation in the *inhA* promoter region [22]. Our result indicated that 35 MDR-TB strains (20.2%) had mutations at codon −15C→T, which was consistent with previous reports. The frequency of other mutations, such as −8T→C, −17G→T and −32G→T, was also found to be same as in previous reports [11, 20]. In total, 72.8% of the INH-resistant strains among the 173 MDR-TB strains studied here had mutations at both katG-315 and inhA −15C→T. This proportion was close to that reported in Shanghai, China (78.5%) [20].

The most common *rpoB* gene mutations observed were at codons 513, 516, 526 and 531. The most frequently mutated codon in our study was codon 531 (41%), this finding being similar to that of a study in Vietnam (37.8%) [24], but lower than that in studies in India [25, 26], Nepal (58.7%) [26] and various other parts of China (58.3–63.3%) [12, 27].

Some studies have reported that *rpoB* gene mutations were responsible for 91–95% of RIF-resistance [13, 20]. In this study, 86.7% of the MDR-TB strains examined here harboured mutations in the RRDR region, which is lower than in previous reports [28–30]. The difference may be due to the following reasons. First, some specimens might contain some inconsistent results between DST and gene sequencing in this study. The possible reason for this was that a solid drug susceptibility test we used in this study, which might not be as sensitive as a liquid drug sensitivity test. There also might be problems of contamination and possible overgrowth with other mycobacteria. Second, the laboratories in this study were limited-resource laboratories, in which laboratory conditions and a lack of practical experience might have influenced the evaluation results. Third, the mutation rate of *rpoB* gene varies greatly among different studies [31], which may be related to the region, sample size or other molecular mechanisms.

Some studies indicated that there may be other drug resistance mechanisms and mutation sites. Mutations associated with RIF resistance can occur in other regions of *rpoB* albeit less frequently (e.g. the V146F mutation in the N-terminal region of *rpoB*) [32]. A study carried out in Africa indicated that Ile491Phe mutations in MDR-TB strains are common [33], and it is thus easy to miss diagnose RIF-resistance using the commercial molecular and phenotypic assays (without containing this mutation) currently endorsed by WHO. We did not find this mutation in our study, but we found a new mutation at Ile572Phe. It will be very important to investigate if other DR-associated mutations are present in other genetic regions and to explore other potential molecular mechanisms that confer resistance in MDR strains. Further molecular analysis of MDR-TB strains that do not contain the well-known mutations will expand our current knowledge of the mechanism conferring drug resistance in the MDR strains.

The Beijing genotypic family *M.tb* is considered one of the most successful genotypes in the present global TB epidemic [19], especially in Eastern Asia. Our data confirm that the Beijing genotype remains the predominant lineage among MDR-TB strains in Beijing. Some studies have suggested that Beijing genotype strains were more likely to be resistant to anti-TB drugs than non-Beijing genotypes [34]. Other studies, however, have indicated that Beijing genotype strains were less likely to acquire drug resistance [12, 15]. Our data are in agreement with the latter finding, we did not find significant differences between Beijing and non-Beijing genotypes.

Our findings did not agree with other reports that Beijing genotype strains have a higher rate of mutations in the *katG* gene [35, 36], nor did our results suggest that Beijing genotype strains have a substantially higher proportion of *rpoB*531 mutations than non-Beijing strains. These results suggest that MDR-TB occurrence is not associated with the prevalence of Beijing genotype strains. It is possible that the association is often influenced by the setting of different projects, drugs types, geographical distribution of the strains [37], the strains chosen or the number of strains included in the study.

## Conclusion

Beijing genotypic MDR-TB strains were spreading in Beijing and present a major challenge to TB control in this region. But they were not significantly associated with the occurrence of drug resistance in Beijing. A high prevalence of katG Ser315Thr, *inhA* promoter region (−15C→T) and *rpoB* (S531L) mutations was observed. Molecular diagnosis based on gene mutations may be a useful method for rapid detection of MDR-TB.

## Data Availability

The online version of this article contains all the data and supplementary material.

## Supplementary material

For supplementary material accompanying this paper visit https://doi.org/10.1017/S0950268820003131. click here to view supplementary material
