# Supplementary material for: Evaluation of the frequency of mutation genes in multidrug-resistant tuberculosis (MDR-TB) strains in Beijing, China
Source: Epidemiol Infect. 2021 Jan 5;149:e21. doi: 10.1017/S0950268820003131 (PMC8057498; doi:10.1017/S0950268820003131)
Supplement: Supplementary file 1 [file S0950268820003131sup001.docx]

Table S1. Differences of characteristics between Beijing and Non-Bejing genotype strains.

| Characteristic | Category | Total | No.of isolates(%) | | | |  |  |
| --- | --- | --- | --- | --- | --- | --- | --- | --- |
|  |  |  | Beijing (n=157) | Non-Beijing (n=16) | OR (95% CI) | *p* value |  |  |
| All |  | 173 |  |  |  |  |  |  |
| sex | Male | 130(75.1) | 120(76.4) | 10(62.5) | 1.223(0.829-1.805) | 0.176 |  |  |
|  | Female | 43(24.9) | 37(23.6) | 6(37.5) |  |  |  |  |
| Age groups, years | <30 | 65 | 60(38.2) | 5(31.3) | 0.223(0.576-2.598) | 0.398 |  |  |
|  | 30-59 | 97 | 88(56.1) | 9(56.3) | 0.996(0.633-1.569) | 0.601 |  |  |
|  | ≥60 | 11 | 9(5.7) | 2(12.6) | 0.459(0.108-1.942) | 0.270 |  |  |
| Status at first episode of tuberculosis | New cases | 93(53.8) | 87(55.4) | 6(37.5) | 1.478(0.773-2.825) | 0.134 |  |  |
|  | Retreatment cases | 80(46.2) | 70(44.6) | 10(62.5) |  |  |  |  |
| residential status | Urban | 101(58.4) | 93(59.2) | 8(50) | 1.185(0.714-1.967) | 0.324 |  |  |
|  | migrant population | 72(41.6) | 64(40.8) | 8(50) |  |  |  |  |

Table S2. Primers used by the PCR method in the hot spots of INH drug target genes

| Gene | Primer | Nucleotide sequence (5'–3') | Annealing temp (°C) | Amplicon position | Product size (bp) | Source or reference |
| --- | --- | --- | --- | --- | --- | --- |
| *katG* | KatG-F1 | 5'ACGAGGCGGAGGTCATCTAC 3' | 60 | 179-846 | 667 | This study |
|  | KatG-R1 | 5'CGAAGCCGAACCCGAACGTC 3' |  |  |  | This study |
|  | KatG-F2 | 5'GCTGCTGTGGCCGGTCAAGA 3' | 62.2 | 727-1342 | 635 | This study |
|  | KatG-R2 | 5'CGTCCTTGGCGGTGTATTGC 3' |  |  |  | This study |
|  | KatG-F3 | 5'GGACGAACACCCCGACGAAA3' | 63 | 1251-1886 | 635 | This study |
|  | KatG-R3 | 5'CGCCGCGGAGTTGAATGACT 3' |  |  |  | This study |
|  | KatG-F4 | 5'TCGGGTGGGAGGTCAACGAC 3' | 61.7 | 1797-2330 | 533 | This study |
|  | KatG-R4 | 5'GCCCTGGTAGGTCCCGTCA3' |  |  |  | This study |
|  | KatG-F5 | 5'TGCTTACGCTCAGTGCCCCT3' | 61.6 | 2136-2677 | 541 | This study |
|  | KatG-R5 | 5'CTCATCCCCGTCTCGTCATC 3' |  |  |  | This study |
| *inhA* promoter region | inhA PR-F1 | 5'CCTCGCTGCCCAGAAAGGGA3' | 60 |  | 248 | This study |
|  | inhA PR-R1 | 5'ATCCCCCGGTTTCCTCCGGT3' |  |  |  | This study |
| *inhA* | inhA-F1 | 5'TCGACGGCGGCATGGGTAT 3' | 62.4 | 155-694 | 559 | This study |
|  | inhA-R1 | 5'ACCGACTCCAACGCGCTCTT3' |  |  |  | This study |
|  | inhA-F2 | 5'CATCCACATCTCGGCGTATT 3' | 61.2 | 558-1089 | 550 | This study |
|  | inhA-R2 | 5'GGCCCCGGGTAACGTTCTC3' |  |  |  | This study |

Table S3. Primers used by the PCR method in the hot spots of RIF drug target genes

| Gene | Primer | Nucleotide sequence (5'–3') | Annealing temp (°C) | Amplicon position | Product size (bp) | Source or reference |
| --- | --- | --- | --- | --- | --- | --- |
| *rpoB* | rpoB-F1 | 5'TAGTTGCGTGCGTGAGATCC3' | 59.2 | 75-656 | 601 | This study |
|  | rpoB-R1 | 5'TCTCGGTCATCATCGGGAAG3' |  |  |  | This study |
|  | rpoB-F2 | 5'CGGCTCCACTGTTCGTCACC3' | 59.6 | 577-1131 | 574 | This study |
|  | rpoB-R2 | 5'CCGAGCTTCTTGTTGACCTT3' |  |  |  | This study |
|  | rpoB-F3 | 5'AGCCCCCGACCAAAGAGTCA3' | 62.3 | 1042-1582 | 557 | This study |
|  | rpoB-R3 | 5'CAGCCCGGCACGCTCAC3' |  |  |  | This study |
|  | rpoB-F4 | 5'CGCTGTCGGGGTTGACCCAC3' | 64.6 | 1522-2014 | 512 | This study |
|  | rpoB-R4 | 5'CGCCTGGCGCTGCATGTTTG3' |  |  |  | This study |
|  | rpoB-F5 | 5'GGACTACATGGACGTCTCGC3' | 61.2 | 1916-2509 | 612 | This study |
|  | rpoB-R5 | 5'ACCTCGTCGGAGATGTTCG3' |  |  |  | This study |
|  | rpoB-F6 | 5'TGTACTGACGACGGCGAGAT3' | 61.1 | 2307-2860 | 571 | This study |
|  | rpoB-R6 | 5'TGCCGATCACGCCCTTGT3' |  |  |  | This study |
|  | rpoB-F7 | 5'CCGCGAGGACGAGGACGAGT3' | 63.8 | 2750-3307 | 577 | This study |
|  | rpoB-R7 | 5'GGTGGAGCGGGCGTGGATCT3' |  |  |  | This study |
|  | rpoB-F8 | 5'GGCGAGCCGTTCCCGTACCC3' | 61.5 | 3234-3808 | 594 | This study |
|  | rpoB-R8 | 5'TGTCCTCCGCGGTAGCAAGA3' |  |  |  | This study |

Table S4. The profiles of Drug Susceptibility Test of those strains.

| No. | Genotype | SITs | Drugs | | | | | | | |
| --- | --- | --- | --- | --- | --- | --- | --- | --- | --- | --- |
|  |  |  | INH | RIF | SM | EMB | LFX | AK | CPM | PAS |
| 1 | Beijing | 1 | R | R | S | R | S | S | S | S |
| 2 | Beijing | 1 | R | R | S | S | S | S | S | S |
| 3 | Beijing | 1 | R | R | R | S | S | S | S | S |
| 4 | Beijing | 1 | R | R | S | S | S | S | S | S |
| 5 | Beijing | 1 | R | R | S | S | S | S | S | S |
| 6 | Beijing | 1 | R | R | R | R | S | S | S | S |
| 7 | Beijing | 1 | R | R | S | S | S | S | S | S |
| 8 | Beijing | 1 | R | R | S | S | S | S | S | S |
| 9 | New found | None | R | R | R | R | R | S | S | S |
| 10 | Beijing | 1 | R | R | R |  | S | S | S | S |
| 11 | Beijing | 1 | R | R | S | S | S | S | S | S |
| 12 | Beijing | 1 | R | R | S | S | S | S | S | S |
| 13 | Beijing | 1 | R | R | R | R | S | S | S | S |
| 14 | Beijing | 1 | R | R | R | R | S | S | S | S |
| 15 | Beijing | 1 | R | R | R | R | S | S | S | S |
| 16 | Beijing | 1 | R | R | R | S | R | S | S | S |
| 17 | Beijing | 1 | R | R | R | S | S | S | S | S |
| 18 | Beijing | 1 | R | R | S | S | S | S | S | S |
| 19 | Beijing | 1 | R | R | R | S | S | S | S | S |
| 20 | Beijing | 1 | R | R | S | S | R | S | S | S |
| 21 | Beijing | 1 | R | R | S | S | S | S | S | S |
| 22 | Beijing | 1 | R | R | R | R | S | R | R | S |
| 23 | Beijing | 1 | R | R | R | R | S | S | S | R |
| 24 | Beijing | 1 | R | R | S | S | S | S | S | S |
| 25 | Beijing | 1 | R | R | S | R | S | S | S | S |
| 26 | Beijing | 1 | R | R | R | S | S | S | S | S |
| 27 | Beijing | 1 | R | R | R | R | S | S | S | S |
| 28 | Beijing | 1 | R | R | S | R | S | S | R | S |
| 29 | Beijing | 1 | R | R | R | S | S | S | S | S |
| 30 | Beijing | 1 | R | R | R | S | S | S | S | S |
| 31 | Beijing | 1 | R | R | R | S | S | S | S | S |
| 32 | MANU2 | 54 | R | R | S | R | R | S | S | R |
| 33 | Beijing | 1 | R | R | S | S | S | S | S | S |
| 34 | Beijing | 1 | R | R | R | R | S | S | S | S |
| 35 | Beijing | 1 | R | R | R | R | S | S | S | S |
| 36 | Beijing | 1 | R | R | R | S | S | S | S | S |
| 37 | Beijing | 1 | R | R | R | R | S | R | S | R |
| 38 | Beijing | 1 | R | R | R | R | S | S | S | S |
| 39 | Beijing | 1 | R | R | R | R | R | R | S | S |
| 40 | Beijing | 1 | R | R | R | R | R | S | S | S |
| 41 | Beijing | 1 | R | R | R | R | R | R | R | R |
| 42 | Beijing | 1 | R | R | R | S | S | S | S | R |
| 43 | Beijing | 1 | R | R | S | R | R | S | S | S |
| 44 | Beijing-Atypical | 190 | R | R | R | R | S | S | S | S |
| 45 | Beijing | 1 | R | R | R | R | R | S | S | S |
| 46 | Beijing | 1 | R | R | R | S | R | S | S | S |
| 47 | Beijing | 1 | R | R | R | R | S | S | S | S |
| 48 | MANU2 | 54 | R | R | R | R | R | R | R | R |
| 49 | T1 | 334 | R | R | S | R | S | S | S | S |
| 50 | Beijing | 1 | R | R | R | S | R | R | S | S |
| 51 | Beijing | 1 | R | R | S | S | S | S | R | S |
| 52 | Beijing | 1 | R | R | R | R | S | S | R | S |
| 53 | Beijing | 1 | R | R | R | R | S | S | S | S |
| 54 | Beijing | 1 | R | R | R | S | S | S | S | S |
| 55 | Beijing | 1 | R | R | R | R | S | S | S | S |
| 56 | Beijing | 1 | R | R | R | S | S | S | S | S |
| 57 | Beijing | 1 | R | R | R | R | S | S | R | S |
| 58 | Beijing | 1 | R | R | S | S | S | S | S | S |
| 59 | Beijing | 1 | R | R | R | R | R | S | S | S |
| 60 | Beijing | 1 | R | R | R | R | S | S | S | S |
| 61 | Beijing | 1 | R | R | R | R | S | S | S | S |
| 62 | Beijing | 1 | R | R | R | R | S | S | S | S |
| 63 | U | 523 | R | R | R | R | S | S | S | S |
| 64 | Beijing | 1 | R | R | R | S | S | S | S | S |
| 65 | T2 | 515 | R | R | S | R | S | S | S | S |
| 66 | Beijing | 1 | R | R | R | S | S | S | S | S |
| 67 | Beijing | 1 | R | R | R | R | S | S | S | S |
| 68 | Beijing | 1 | R | R | R | R | R | S | S | S |
| 69 | Beijing | 1 | R | R | R | R | S | S | S | S |
| 70 | Beijing | 1 | R | R | S | R | S | S | S | S |
| 71 | Beijing | 1 | R | R | R | R | S | S | S | S |
| 72 | Beijing | 1 | R | R | R | R | R | S | S | S |
| 73 | Beijing | 1 | R | R | R | S | S | S | S | S |
| 74 | Beijing | 1 | R | R | R | S | R | S | S | S |
| 75 | Beijing | 1 | R | R | R | R | S | R | S | S |
| 76 | Beijing | 1 | R | R | R | R | R | S | S | S |
| 77 | Beijing | 1 | R | R | S | S | R | R | S | S |
| 78 | Beijing | 1 | R | R | R | R | S | S | S | S |
| 79 | MANU2 | 1192 | R | R | R | R | R | S | S | S |
| 80 | Beijing | 1 | R | R | S | R | S | S | S | S |
| 81 | Beijing | 1 | R | R | R | R | S | R | R | S |
| 82 | Beijing | 1 | R | R | R | R | S | S | S | S |
| 83 | Beijing | 1 | R | R | R | S | S | S | S | S |
| 84 | Beijing | 1 | R | R | R | R | S | S | S | S |
| 85 | Beijing- Atypical | None | R | R | S | R | S | S | S | S |
| 86 | Beijing | 1 | R | R | S | R | S | S | S | S |
| 87 | Beijing | 1 | R | R | R | R | R | R | R | R |
| 88 | Beijing | 1 | R | R | R | R | S | S | S | S |
| 89 | Beijing | 1 | R | R | S | S | R | S | S | S |
| 90 | Beijing | 1 | R | R | R | R | R | R | S | S |
| 91 | Beijing | 1 | R | R | R | S | R | S | S | S |
| 92 | Beijing | 1 | R | R | R | R | S | S | S | S |
| 93 | Beijing | 1 | R | R | R | R | S | S | S | S |
| 94 | Beijing | 1 | R | R | R | R | R | S | S | S |
| 95 | Beijing | 1 | R | R | R | R | R | S | S | S |
| 96 | Beijing | 1 | R | R | R | R | R | S | S | S |
| 97 | Beijing | 1 | R | R | R | R | S | S | S | S |
| 98 | Beijing | 1 | R | R | R | R | R | S | S | R |
| 99 | MANU2 | 1088 | R | R | R | R | S | S | S | S |
| 100 | New found | None | R | R | S | R | S | S | S | S |
| 101 | Beijing | 1 | R | R | R | R | R | S | S | R |
| 102 | Beijing | 1 | R | R | R | R | R | S | S | S |
| 103 | Beijing | 1 | R | R | R | R | S | S | S | S |
| 104 | Beijing | 1 | R | R | S | S | R | R | S | S |
| 105 | Beijing | 1 | R | R | R | S | S | S | S | S |
| 106 | Beijing | 1 | R | R | S | R | S | S | S | S |
| 107 | Beijing | 1 | R | R | R | R | R | R | R | R |
| 108 | Beijing | 1 | R | R | S | R | R | S | S | S |
| 109 | Beijing | 1 | R | R | R | R | S | S | S | S |
| 110 | T1 | 803 | R | R | S | S | S | S | S | S |
| 111 | Beijing | 1 | R | R | R | S | S | S | S | S |
| 112 | Beijing | 1 | R | R | R | R | R | S | S | R |
| 113 | Beijing | 1 | R | R | R | R | R | S | S | S |
| 114 | Beijing | 1 | R | R | S | R | R | S | S | S |
| 115 | Beijing | 1 | R | R | R | R | S | S | S | S |
| 116 | Beijing | 1 | R | R | R | R | R |  |  |  |
| 117 | Beijing | 1 | R | R | R | R | R | R | R | R |
| 118 | Beijing | 1 | R | R | R | R | R | S | S | S |
| 119 | Beijing | 1 | R | R | R | R | R | R | R | S |
| 120 | Beijing | 1 | R | R | S | S | S | S | S | S |
| 121 | Beijing | 1 | R | R | R | R | R | R | R | S |
| 122 | Beijing | 1 | R | R | R | S | S | S | S | S |
| 123 | Beijing | 1 | R | R | R | R | S | S | S | S |
| 124 | Beijing | 1 | R | R | S | R | S | S | S | S |
| 125 | Beijing | 1 | R | R | R | S | S | S | S | R |
| 126 | Beijing | 1 | R | R | R | R | S | S | S | R |
| 127 | Beijing | 1 | R | R | R | R | R | S | S | S |
| 128 | T1 | 154 | R | R | S | S | S | S | S | S |
| 129 | Beijing | 1 | R | R | R | R | R | S | S | S |
| 130 | Beijing | 1 | R | R | S | S | S | S | S | S |
| 131 | Beijing | 1 | R | R | S | R | S | S | S | S |
| 132 | Beijing | 1 | R | R | R | R | S | S | S | S |
| 133 | Beijing | 1 | R | R | R | R | R | R | R | S |
| 134 | Beijing | 1 | R | R | R | R | S | S | S | R |
| 135 | Beijing | 1 | R | R | R | R | R | S | S | S |
| 136 | Beijing | 1 | R | R | R | R | S | S | S | S |
| 137 | Beijing | 1 | R | R | R | R | S | S | S | S |
| 138 | Beijing | 1 | R | R | R | R | S | S | S | S |
| 139 | Beijing | 1 | R | R | R | R | R | R | R | R |
| 140 | Beijing | 1 | R | R | S | S | S | S | S | S |
| 141 | Manu2 | 1247 | R | R | S | S | S | S | S | S |
| 142 | Beijing | 1 | R | R | R | R | R | S | S | S |
| 143 | Beijing | 1 | R | R | R | R | S | S | S | S |
| 144 | Manu ancester | 523 | R | R | S | S | S | S | S | S |
| 145 | Beijing | 1 | R | R | R | R | S | S | S | S |
| 146 | Beijing | 1 | R | R | R | R | R | S | S | S |
| 147 | Beijing | 1 | R | R | R | R | S | R | R | S |
| 148 | Beijing | 1 | R | R | R | R | S | S | S | S |
| 149 | Beijing | 1 | R | R | R | R | S | S | S | S |
| 150 | Beijing | 1 | R | R | R | R | S | S | S | S |
| 151 | Beijing | 1 | R | R | R | S | S | S | S | S |
| 152 | Beijing | 1 | R | R | S | S | S | S | S | S |
| 153 | Beijing | 1 | R | R | R | R | S | S | S | S |
| 154 | Beijing | 1 | R | R | R | R | S | S | S | S |
| 155 | Beijing- Atypical | 2610 | R | R | R | R | R | S | S | S |
| 156 | Beijing | 1 | R | R | R | R | S | S | S | S |
| 157 | Beijing- Atypical | 2610 | R | R | R | R | S | S | S | S |
| 158 | Beijing | 1 | R | R | S | S | R | S | S | S |
| 159 | Beijing | 1 | R | R | S | R | S | S | S | S |
| 160 | Beijing | 1 | R | R | R | R | S | S | S | S |
| 161 | Beijing | 1 | R | R | R | R | S | R | S | S |
| 162 | MANU2 | 54 | R | R | R | R | R | S | S | S |
| 163 | Beijing | 1 | R | R | R | S | R | S | S | S |
| 164 | Beijing | 1 | R | R | R | R | S | S | R | S |
| 165 | MANU2 | 54 | R | R | S | R | R | S | S | S |
| 166 | Beijing | 1 | R | R | R | S | S | S | S | S |
| 167 | T2 | None | R | R | R | S | S | S | S | S |
| 168 | Beijing- Atypical | None | R | R | S | S | S | S | S | S |
| 169 | Beijing | 1 | R | R | R | R | S | S | S | R |
| 170 | Beijing | 1 | R | R | R | R | S | S | S | S |
| 171 | Beijing | 1 | R | R | R | R | R | S | S | S |
| 172 | Beijing | 1 | R | R | R | R | S | S | S | S |
| 173 | Beijing | 1 | R | R | R | S | S | S | S | S |

INH: Isoniazid; RIF: Rifampicin; SM: Streptomycin; EMB: Ethambutol; LFX: Levofloxacin; AK: amikacin; CPM: capreomycin; PAS: p-aminosalicylic acid.
